# *In vivo* photopharmacological inhibition of hippocampal activity *via* multimodal probes – perspective and opening steps on experimental and computational challenges†

**DOI:** 10.1039/d4tb01117a

**Published:** 2024-08-15

**Authors:** Johannes Gurke, Alejandro Carnicer-Lombarte, Tobias E. Naegele, Anders K. Hansen, George G. Malliaras

**Affiliations:** a University of Potsdam, Institute of Chemistry Karl-Liebknecht-Str. 24-25 14476 Potsdam Germany johannes.gurke@uni-potsdam.de; b University of Cambridge, Electrical Engineering Division 9 JJ Thomson Ave Cambridge CB3 0FA UK; c Fraunhofer Institute of Applied Polymer Research (IAP) Geiselbergstraße 69 14476 Potsdam Germany; d Technical University of Denmark, DTU Fotonik Frederiksborgvej 399 4000 Roskilde Denmark

## Abstract

Neurological conditions such as epilepsy can have a significant impact on people's lives. Here, we discuss a new perspective for the study/treatment of these conditions using photopharmacology. A multimodal, intracranial implant that incorporates fluidic channels for localised drug delivery, electrodes for recording and stimulation, and a light source for photoswitching is used for *in vivo* administration and deactivation of a photoresponsive AMPA antagonist. We review current advancements in the relevant disciplines and show experimentally that the inhibition of seizure-like events induced in the hippocampus by electrical stimulation can be altered upon switching the drug with light. We discuss the interconnection of the drug's photopharmacological properties with the design of the device by modelling light penetration into the rat brain with Monte Carlo simulations. This work delivers a new perspective, including initial experimental and computational efforts on *in vivo* photopharmacology to understand and eventually treat neurological conditions.

## Abstract 2

A new perspective for the study/treatment of neurological conditions using *in vivo* photopharmacology is presented, including initial experimental and computational data.

## Graphical abstract

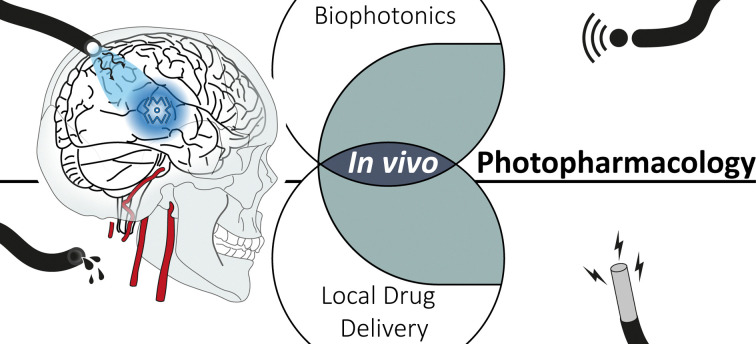

## Introduction

The dysfunction of hippocampal neurological circuits is a pressing issue, leading to severe neurological conditions that significantly impact patients’ lives.^1^ Memory disorders^2,3^ and epilepsy^4^ are just two examples. The imbalance between excitatory and inhibitory activity, caused by, *e.g.* overexpression of glutamate, is credited to be the origin of epileptic seizures.^5^ Glutamate overexpression can lead to neuronal excitotoxicity.^6^ Excitotoxicity in epilepsy is associated with neurodegeneration (death of neurons), and is hypothesised to be a factor in some neurodegenerative diseases. Therefore, it is crucial to develop local and rapid interventions to prevent long-term effects and manage short-term symptoms caused by abnormal neuronal activity.^7,8^ An option is the systemic administration of anticonvulsant medication. Not only is this prone to cause grave side effects but, for the example of epilepsy, *ca.* 30% of patients show pharmacoresistance.^9,10^ Local drug delivery is under intense preclinical investigation, holding great potential for a precise yet mostly invasive treatment. This can be achieved using various methods and delivery mechanisms, *e.g.*, convection-enhanced *via* a (micro)fluidic system;^11–13^ electro- or iontophoretically, where an electric field drives charged drugs;^14–20^ or passively by diffusion out of a systemically administered or locally implanted carrier.^21,22^ Two examples of a local administration of inhibitory agents are the local release of GABA,^14,15^ the universal inhibitory neurotransmitter *via* electrophoretic “dry” drug delivery or the convection-enhanced delivery of MPQX, and glutamate antagonist for the AMPA receptor.^23^ Aside from pharmacological *in vivo* neuromodulation, other modes of action are being investigated. Externally induced excitatory actions on neurological circuits are well-studied, while inhibitory actions are more challenging. Electrical stimulation protocols have been investigated to induce inhibition in the rat cortex.^24^ Deep brain stimulation is famously used to inhibit dysfunctional circuits causing tremors and Parkinson's disease.^25,26^ Temporal interference can be used to achieve deep brain stimulation within the hippocampus (HC).^27–30^

Pharmacological inhibition is highly precise in a biochemical sense, as specific molecular targets are addressed. However, it lacks pace and on-demand controllability, as observed in the example of Proctor *et al.*^14,15^ Between the initiation of the delivery and a visible therapeutic effect in the electrophysiological signal, up to 60 s can pass due to diffusion from the implant side into the targeted tissue. Moreover, local overdosing can still be an issue. Electric neuromodulation methods are far more rapid^31^ yet less selective, and overstimulation and severe tissue damage are possible.^31^ Optogenetics uses light as a trigger through cells with photoresponsive transmembrane proteins.^32–35^ It allows the most rapid neuromodulation, yet these photoresponsive proteins do not naturally occur in vertebrates. The expression requires the treatment of the target tissue with a gene vector prior to the modulation.

Photopharmacology utilises reversible photoswitches incorporated into the drug's chemical structure to alter its pharmacological effect. An *in vivo* application presents a promising alternative for neuromodulation, surpassing the aforementioned limitations.^36–41^ Photopharmacological agents’ preparation, administration, characterisation and transfer into a clinic application can be conducted analogously to “normal” (not photoresponsive) drugs through well-established processes.

**Scheme 1:**
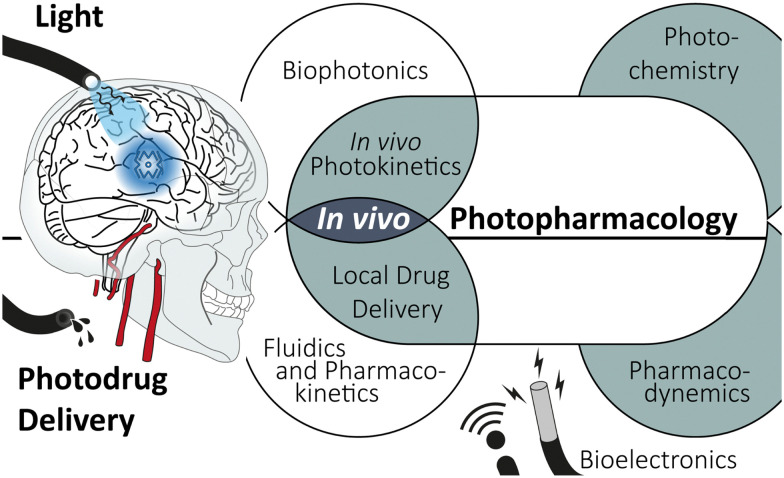
*In vivo* photopharmacology, an interdisciplinary endeavour.

Transferring photodrugs from the *in vitro* experiment to the *in vivo* environment is a highly interdisciplinary endeavour (see Scheme 1). Organic synthesis, photochemistry, and pharmacological aspects must be paired with medical device engineering, including bioelectronics and photonics, as well as local drug delivery. The incorporation of multiple functions, namely (fluidic) drug administration, electrophoretic recording and light illumination, into a single, concise implant is an ongoing engineering challenge. These so-called multimodul probes are essential tools for applying photopharmacology.

Here, we give a concise overview of photopharmacology, summarise the cutting-edge advancements in device technology, and offer a new perspective on the *in vivo* application of photodrugs. Initial experimental and computational findings will substantiate this perspective. Lastly, we outlay a path towards an overarching model. This computational bridging of instrumentation and photochemical properties opens a pathway to streamline device design and photodrug application.

## Current state & challenges in photopharmacology

**Fig. 1:**
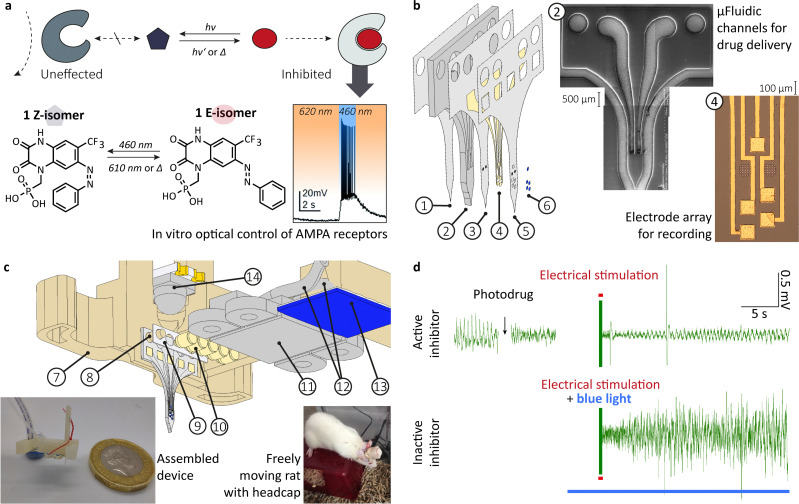
From *in vitro* optical control of AMPA receptor to *in vivo* photopharmacological inhibition of hippocampal activity. (a) schema depicting the mode of action, chemical structure and *in vitro* optical control of action potential firing in hippocampal CA1 neurons using 1 in the presence of excitatory neurotransmitter glutamate; (b) exploded-view drawing of a multilayerd probe design ((1) bottom PAC layer (2 μm), (2) 3D printed, flexible body with microfluidic system (200 μm), (3) middle PAC layer (2 μm) with μ-fluidic in- and outlet, (4) 10 nm titanium-100 nm gold electrodes (5), top PAC layer (2 μm) with etched μ-fluidic in- and outlets as well as electrical contacts, (6) Drop-on-demand inkjet-printed PEDOT:PSS layer (77 ± 11 nm)), a SEM pictures of the printed μ-fluidics and a microscopy picture of the gold electrode array; (c) exploded-view drawing of the assembled device incorporating (14) a bi-colour SMD-LED chip with dome lens, (7) 3D printed socket with (8) integrated alignment pins, (9) medical-grade double-sided adhesive tape, (10) pogo pins (85 μm), (11) 3D printed fluidic and electric interface with M0.6 thread, (12) 1/32′′ polyethylene tubing (0.8 mm OD, 0.4 mm ID) and (13) tailored FPC cable (5 ways, 1 mm pitch) left insert shows a picture of the assembled device with a one GBP coin for scale and the right insert show a freely moving rat with implanted device and headcap; (d) under anaesthesia *in vivo* rodent models to conceptually prove the photopharmacological inhibition of hippocampal activity, using the multimodal implant. Top: Neural recording from rat HC, showing decreasing amplitude of interictal-like spiking activity after drug administration (*n* = 1). In response to electrical stimulation (red line) after delivery of the active photodrug, neural recording shows minor interictal-like spiking, indicating successful suppression by the photodrug. Bottom: The delivery of active photodrug is followed by blue light illumination prior to electrical stimulation. The stimulation in the presence of the inactive photodrug leads to large amplitude interictal-like spiking activity. (a) and (b) as well as the inserts in (c) were reproduced from ref. 23 and 47 with permission from the Royal Society of Chemistry.

Numerous modes of intervention with biological processes by photochemical means have been studied. Besides optogenetics, where proteins are intrinsically photoresponsive, and photodynamic therapy,^42,43^ which uses the photogeneration of reactive oxygen species, other modes of action are investigated. Photophamacological effects are achieved by irreversible photouncaging and reversible photoactivation, to name just two. The latter uses photochromic ligands (PCLs), which undergo reversible photoreactions (photoswitching) between two (or more) forms, with each form having a different absorption spectrum. Depending on the form, the PCLs have a higher or lower affinity to the binding site of the biological target, i.g., in a host–guest interaction (see Fig. 1a). Trauner and coworkers not only provided a comprehensive review of molecular advances in the field but also pioneered photopharmacology targeting neurologically relevant targets.^39,44–51^

A variety of structural motifs are under investigation for photopharmacological applications;^39^ among others azobenzenes, bistable diarylethene and hemithioindigo photoswitches. The azobenzene motif is the most used structure among the molecular photoswitches. It undergoes an *E* ⇌ *Z* isomerisation upon light illumination. The change in pharmacodynamic properties upon the photoreaction is primarily allocated to the shift in steric demand and, consequently, in host–guest interactions with a target. However, a change in the dipole moment amongst the two isomers can also affect intermolecular interactions. The *E*-isomer commonly responds to shorter wavelengths, while the *Z*-isomer exhibits a bathochromic shift in its absorption spectrum. Generally speaking, a strong red shift for both photoreactions is desirable, as the light penetration depth into organ tissue is strongly increased for longer wavelengths. Thus, the target of ongoing research efforts is an (infra)red photochromic compound without impairment of the photoswitching performance.^52–61^ Ideally, a new chemical motif is synthetically easily accessible and as universally applicable as the azobenzene.^62^*ortho* substitutions of azobenzene moieties with oxy groups are one example studied, achieving a strong bathochromic shift while preserving good switching properties.^63,64^ Alternatively, two-photon absorption, up-conversion or radioluminescent nanomaterials are under investigation to bypass a limited light penetration depth.^65–68^

The *E*-isomer is, besides particular substitution patterns, the thermodynamically favoured form. A thermal interconversion back to the *E*-isomer strongly depends on the substitution pattern and can be tuned, ranging from a few milliseconds to years in terms of the half-life time (*t*_1/2_).^69,70^ Fluorine substituents are known to prolong the half-life,^71–73^ while push–pull motives shorten it substantially.^59,74^ An ideal reaction rate of the thermal backreaction strongly depends on the contemplated application. Reductive milieus can cause conversion of the azobenzene (–N=N–) to hydrazine (–HN–HN–),^75,76^ interfering with the thermal stability. Electron-withdrawing substituents, like fluorine, increase the electron affinity and, hence, decrease the stability towards this reduction. Intramolecular hydrogen bonds to the nitrogens of the azobenzene motif are also known to change thermal stability.^72^ The chemical equilibrium of a drug binding to its target largely defines its pharmacodynamic properties. The difference in this binding among two photoisomers determines the alteration in the pharmacodynamic properties. If host–guest interactions influence the photochemical reactions, i.g., if the bind azobenzene undergoes a *Z* → *E* isomerisation or if only the free molecule converts back, is under debate.^77–79^

Optogenetic as well as photophamacological systems can be characterised by their light responsiveness (the wavelength-dependent product of the molar extinction coefficient *ε*_*i*_(*λ*) of the photoresponsive entity *i* and quantum yield *φ*_*ij*_(*λ*) of a photophysical or photochemical process *ij*).^80–85^ A large spectral difference among the *E*- and *Z*-isomers is desirable, as it is one way to alter light responsiveness significantly. An ideal system has two distinctly addressable wavelengths at which either *E* → *Z* or *Z* → *E* isomerisation can be conducted:

1 *ε*_*E*_(*λ*)·*φ*_*EZ*_(*λ*) ≠ 0 and *ε*_*Z*_(*λ*)·*φ*_*ZE*_(*λ*) = 0;

or

2 *ε*_*E*_(*λ*)·φ_*EZ*_(*λ*) ≫ *ε*_*Z*_(*λ*)·*φ*_*ZE*_(*λ*)

where *ε*_*E*_(*λ*) and *ε*_*Z*_(*λ*) are the molar extinction coefficient of the *E*- and *Z*-isomer, respectively; *φ*_*EZ*_(*λ*) and *φ*_*ZE*_(*λ*) the quantum yield of the photoisomerisations is at a given exposure wavelength. In this ideal case, light exposure results in a complete conversion. As a system diverges from condition (2), a mixture of isomers is observed at the photostationary state. When a fast thermal interconversion is present, the light responsiveness is insufficient to characterise the system comprehensively. The photon flux (proportional to the irradiance) must be included, as the thermal and photochemical reaction rates must be put into relation to each other. Hence, detailed information about the photochemical properties of the PCLs and of the local irradiance must be included for a comprehensive description of a photophamacological system.

We used the photodrug 1 (Fig. 1a) for *in vivo* photopharmacological inhibition of hippocampal activity. The photodrug has been develop and characterised *in vitro* by Trauner and coworker.^47^ It is an AMPA antagonist in its *E*-isomer. It inhibits the signal transfer in the dark or upon irradiation at 620 nm. The irradiation with 460 nm permits action potential firing in hippocampal CA1 neurons when glutamate is present.

A thermal backreaction occurs in the dark, leading to a significant increase in the antagonism. A half maximal inhibitory concentration IC_50_ of 3.1 μM was determined in the dark, which is a little less potent than its non-photoswitchable analogue, Fanapanel (also MPQX, 0.12 μM).^86^

Compound 1 is inhibiting in its thermodynamically stable *Z*-isomer, which is a major drawback. The pharmacological effect is deactivated by light, demanding constant irradiation to control action potentials precisely. Rephrasing the inhibiting effect of 1 is active by default. A so-called signal inversion has been explored for a few other photodrugs using bridged azobenzenes where the *E*-isomer is the thermodynamically stable form.^87^ Applied to compound 1 in a new derivative would allow an inactive default state.

PCL 1 shows a change in the absorption spectrum when mixed with l-arginine. This reinforces the general question of whether the *ex vivo*-determined photochemical properties (*ε*_*i*_(*λ*), *φ*_*ZE*_(*λ*), and *t*_1/2_) are valid in the *in vivo* environment.

## Multimodal implants

Multimodal penetrating brain probes allow simultaneous functionality such as light exposure, electrophysiological recording, electric stimulation, electrochemical sensing and drug delivery.^37,40,88–90^ Various reviews on this topic have been published, *e.g.* by Qazi *et al.*^91^ They show general and ongoing research objectives among all modules concerning miniaturisation, wireless data and energy transfer, chronic biocompatibility and long-term device performance, and scaling in fabrication. Recently, the operation of multimodal implants has been demonstrated in unrestrained animals using head-mounted and fully implanted approaches.

### Light delivery

The momentum in optogenetics led to a wide range of setups to illuminate the target tissue,^92–96^ where miniaturisation was a driving force. Early setups used the combination of an external light source, *e.g.* a laser with optical fibres or other wave-guiding approaches. Incorporating this with a measuring electrode, the first optrode was developed in 2007 and designed for simultaneous irradiation and read-out.^97^ The latest generation incorporates surface-mount device (SMD)- micro light emitting diodes (μLED) into the tip of the probes.^98–100^ Although more challenging fabrication-wise, these setups increases the power output at the targeted tissue. However, heat dissipation into the tissue has to be considered. Mounting the stiff μLED on flexible substrates allowed for mechanical matching to soft tissues, like the brain, and increased compatibility accordingly.^101^ Furthermore, the mounted LEDs require encapsulation to ensure long lifetimes and biocompatibility.^102,103^ Organic LEDs have been explored as flexible, large-area light sources.^104–106^ However, power output and lifetime are still unfavourable compared to LEDs, preventing their broader application.

### Recording and sensing

The integration of a recording site allows for a closed-loop operation mode.^107–109^ It describes the adjustment of a neural modulation or treatment to electrophysiological measurements or chemical sensing. Plastic electronics is having a substantial impact. It uses nanometer-thin metal films on a polymer substrate and organic conductive materials like conjugated polymers or graphene, achieving flexible devices with improved signal-to-noise ratios.^110^ PEDOT:PSS-coated gold electrodes have become the gold standard in that regard, as they show a reduced impedance in contact with aqueous media, which is highly beneficial for electrophysiological recordings. Ongoing efforts are made to develop improved materials. Organic electrochemical transistors (OECTs) are laborious to manufacture and require more elaborate instrumentation while recording yet yield even better signal-to-noise ratios.^111,112^ Furthermore, they can be functionalised for biochemical sensing,^113,114^*e.g.*, sodium ions,^115^ dopamine,^116^ cortisol,^117^ amyloid-β,^118^ and more.

The current challenges are long-term stability and avoiding delamination under chemical or mechanical stress.^119–122^ Here, the metal layers on polymer substrates seem to be a weak point, while organic materials show better adhesion and potentially allow for a covalent attachment.

### Drug delivery

Local drug delivery systems mainly make use of convection-enhanced delivery.^11–13^ Significant efforts focused on miniaturisation and approval of medical devices of the drug pump and reservoir. Convection-enhanced delivery has several drawbacks, *e.g.* unintended, constant leakage of drug and increasing intracranial pressure throughout the injection. To overcome the drawbacks of convection-enhanced delivery, alternative delivery mechanisms are under development, *e.g.* by electrical means.^14–20^ Choosing an appropriate ion-selective membrane material fitting to the delivered drug and ideal electrical operation protocols are ongoing challenges.^123,124^ To avoid an electrochemical reaction of the drug at the working electrode, *e.g.* the previously discussed reduction of the azobenzene, and to achieve a constant drug output, the separation of the drug solution and the working electrode is beneficial. We developed a redox flow iontophoresis for continuous drug delivery, until now, for cationic drugs.^20^ The technology must be adopted for anions, as photodrug **1** is, and then be transferred into a multimodal probe in future.

### Multimodal probe for *in vivo* photopharmacological inhibition of hippocampal activity

Currently, considerable efforts are being made to develop scalable fabrication methods that will greatly impact larger numbers of patients. This includes standardised manufacturing protocols and uniform devices. Dividing from this, we see anatomic or patient-specific implants as a promising approach. It will fully use computer-aided design at a digital twin of the patient and subsequential computer-aided manufacturing.^125,126^ Hence, we explored 3D printing as a manufacturing technique and how to combine it with classic microfabrication to create a hybrid fabrication scheme. To administer a drug, record and stimulate neural activity, we previously designed and fabricated a multimodal probe (Fig. 1b), followed by the heterointegration into a device for rodent model (Fig. 1c).^23^

We extended our previously developed device. A simple butt-coupling of a bi-colour SMD-LED, integrated in the headstage allowed for effective illumination.‡‡Original research data first published here. We calculated a power output of 58.5 ± 0.2 mW (460 nm) and 33.6 ± 0.2 mW (610 nm).^127^ Multiday implantations have been conducted into freely moving rats, although actual experiments still require a tether.^128^

## Perspective on the application of *in vivo* photopharmacological inhibition

The research on pharmacoresistant epilepsy aims for an enhancement in the success of the treatment and minimisation of side effects. Measuring the hippocampal activity coupled to an intracranial drug injection, inhibiting action potential firing, is a potent way to reach those aims. Even though a precise measurement of brain activity is possible, the exact prediction of both the seizure's occurrence and intensity is still out of reach. This fact makes a high temporal control over the antiseizure drug concentration in the target area vital to ensure treatment can be delivered with minimal latency following seizure detection. The diffusion of the drug strongly limits the existing technologies. Using a photopharmacological agent which can be injected in its inactive form and reversibly activated on demand has a vast potential to bypass this problem and improve epilepsy treatment. Especially closing the loop with simultaneous electrophoretic measurement of neuronal activities will allow a timely adjustment of active drug concentration to the actual seizure intensity.

In a first study, we showed the application of the non-photoswitchable derivative, MPQX.^23^ For that purpose, an implantation protocol of the built multimodal probe for an acute drug administration into a rat's HC has been established. Both recordings of hippocampal activity, as well as electrical stimulation, were conducted using the gold-PEDOT:PSS electrode array. Electrical stimulation of the naive rat brain produced large amplitude interictal-like spikes. Subsequent to MPQX administration, electrical stimulation resulted in a significantly lower number of interictal spike development compared to naïve brain stimulation.

Here, we show original data, extending the first study.‡ We examined the administration of photodrug 1 (Fig. 1a), using an upgraded probe (with light source, Fig. 1c) and the same protocol for the *in vivo* experiments. In its dark (active) state, electrical stimulation only caused low levels of interictal spiking (Fig. 1d top, and Fig. S2, ESI†). When illuminated with 460 nm before the stimulation to inactivate the photodrug, the stimulation led to higher amounts of activity (Fig. 1d bottom and Fig. S2, ESI†). In the absence of photodrug 1, blue light alone has no effect in seizure prevention (Fig. S3, ESI†). This experiment is the first indication of the validity of the *in vivo* inhibition of seizure-like events using photodrug **1**. Future studies will aim to reproduce the *in vivo* experiments for larger amounts of rodents. Moreover, we aim to extend our *in vivo* protocols towards spontaneous evolving seizure-like events, *e.g.* through injection of 4-aminopyridine and their counteracting. This will allow a comparison with traditional treatment methods, *e.g.* resective epilepsy surgery for drug-resistant epilepsy.

Besides the short-term objective to solidify the experimental proof for an *in vivo* photopharmacological inhibition, medium- and long-term objectives must be addressed. For instance, a new derivative of compound 1 must be developed, which copes with the shortcoming of a drug active by default. For a reasonable application, the photoresponsive AMPA antagonist should be inactive by default and activated by light. Such a photodrug, combined with constant monitoring of neural activity, will enable a new perspective for the treatment of focal, drug-resistant epilepsy. The recording in the target brain area will allow predicting the likelihood of a seizure. Suppose the forecast indicates an increased likelihood of an epileptic event, i.g., due to detecting a specific signal structure, the inactive photoresponsive AMPA antagonists will be injected, not affecting the neural activity. In case of an actual seizure, the photodrug will be irradiated on demand, suppressing active potential firing within milliseconds. Depending on the measured intensity of the seizure, the light power and ratio of activating and deactivating light will be modulated, affording precise control. The reversibility of the photoswitch enables control over the dose and deactivation within the tissue after the pathologic event. This will prevent overdosage and minimise side effects. Computational (AI) methods could optimise such a monitoring → injection → detection → activation ⇌ deactivation loop. Applied to other brain regions, such a concept could be used in conditions other than epilepsy. Examples of this may be the study of neuronal circuit function through fast pharmacological intervention (*e.g.* deactivating certain populations of neurons during a behavioural task), or the treatment of conditions benefiting from time-dependent or closed-loop delivery of therapy such as depression and chronic pain.^129,130^ The fast and controlled pharmacological intervention enabled by this technology holds great potential to both enable new treatments and decrease dosage and side effects of existing ones.

## Connecting engineering with chemistry

Rationalising device engineering and understanding the fundamental behaviour of the photodrug within the tissue requires the interconnection of multiple disciplines. *In vivo* photopharmacology, on its own, leaving bioelectronic recording and stimulation at a side involves the interconnection of microfluidics and pharmacokinetics, including drug diffusion within the tissue. Moreover, it requires an understanding of light propagation within the biological tissue and the photokinetics of the photodrug.

Computational modelling will give access to the most relevant insights for the application of existing compounds: What area can be treated? What light power output is required to reduce thermal strain? How can the active drug concentration be controlled? What is the response time? Moreover, it can also loop back into new photodrug development: What photo- and pharmacodynamic properties are actually required for a particular application?

**Scheme 2:**
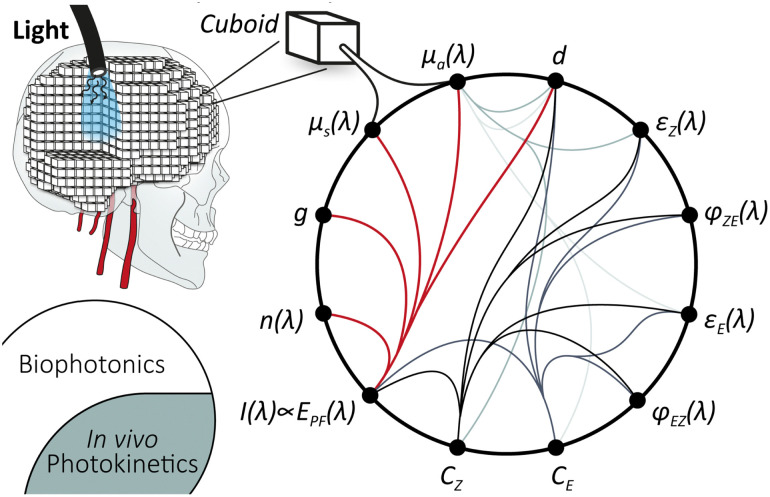
Toward *in vivo* photokinetic modelling *via* Monte Carlo simulation, involving a variety of interwoven variables and experimental values: absorption coefficient *μ*_a_(*λ*) [cm^−1^], scattering coefficient *μ*_s_(*λ*) [cm^−1^], Henyey–Greenstein scattering anisotropy factor *g*, refractive index *n*(*λ*), irradiance *I*(*λ*) [W cm^−2^] ∝ photon flux *E*_PF_(*λ*) [E], molar absorption coefficients *ε*_*E*_(*λ*) and *ε*_*Z*_(*λ*) [L mol^−1^ cm^−1^], quantum yield *φ*_*ZE*_(*λ*) and *φ*_*EZ*_(*λ*), path length *d*, and concentration *C*_*E*_ and *C*_*Z*_. (*λ*) represent a wavelength-dependent function.

Monte Carlo simulations hold the potential for comprehensive modelling of all relevant processes when fed with sufficient experimental values. Both drug diffusion^131^ and light propagation in highly scattering media^132,133^ have been studied. The starting point is the combination of photochemistry with biophotonics into an *in vivo* photokinetic model. Such a concept has already been discussed in photodynamic therapy.^134^ For the Monte Carlo simulation, a section of the target tissue is divided into individual cuboids of defined size, holding the physical properties (see Scheme 2). For light propagation, this includes absorption coefficient *μ*_a_, scattering coefficient *μ*_s_, Henyey–Greenstein scattering anisotropy factor *g*, and refractive index *n*(*λ*), extensively summarised by Prof. Jacques.^135^

### High-resolution MC modelling of light delivery in a rat brain‡

**Fig. 2:**
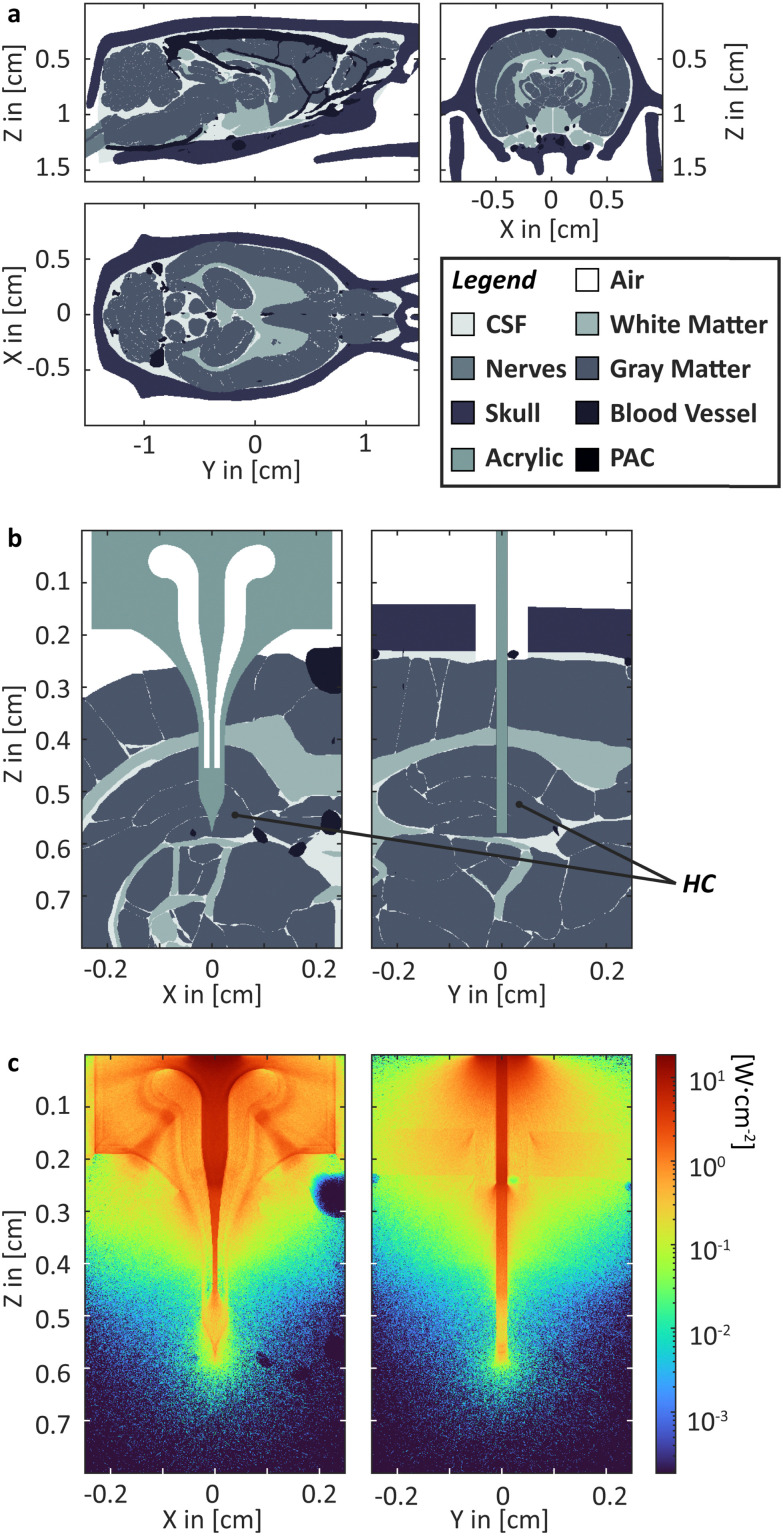
MS Simulation. (a) Rat head model segmented into six different kinds of tissue; (b) head section and probe in HC with; (c) heatmap depiction of the irradiance within the head section for 460 nm.‡

For our purpose, an anatomic 3D model of a rat brain with the target tissue is required. Comprehensive, high-quality segmented 3D data, including all relevant tissue types, are currently unavailable. Hence, we combined data from three different MRI studies to generate a model, including white and grey matter, blood vessels, nerve tissue, cerebrospinal fluid (CSF), and bone (see Fig. 2a).^136–138^ The resulting model must be seen as a rough estimation. Various digital artefacts are present, *e.g.*, lines between brain regions originating from the digital segmentation or misfitting of the inferior vascular system. A digital model of the probe, including the printed acrylic body and PAC layers, has been placed, penetrating the HC (Fig. 2b). Parts of the scull have been digitally removed, and a butt-coupled LED light source has been simulated. High-resolution modelling is required as the PAC layers are just 4 μm thick, and a sufficiently large area must be considered to make a useful simulation. Accordingly, a section of 0.4 × 0.4 × 0.8 cm^3^ with cuboids 4 × 4 × 8 μm^3^ was used, resulting in a matrix with 1.56 × 10^9^ elements. A high-performance computer cluster was used to calculate *ca.* 7.9 × 10^6^ photons per wavelength over 10 min simulation time. As expected, red light penetrates deeply into the tissue, not requiring a waveguide (see supporting information Fig. S1, ESI†). Blue light strongly benefits from the waveguide. Without the probe, delivering the light, an illumination of the HC is not feasible.

### Towards *in vivo* photokinetic model

Azobenzene switching is a photochemical equilibrium between the *E* and *Z* isomers. The final equilibrium *K*_*EZ*_ at the photostationary state and the time to reach it depend on molecular properties and the used wavelength. As azobenzenes, including the used photodrug 1, are T-type photoswitches, both photochemical and thermal interconversion must be considered.3
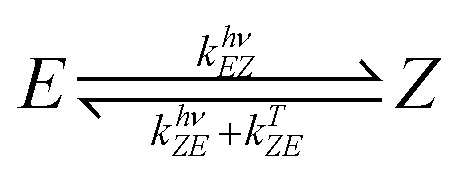
4
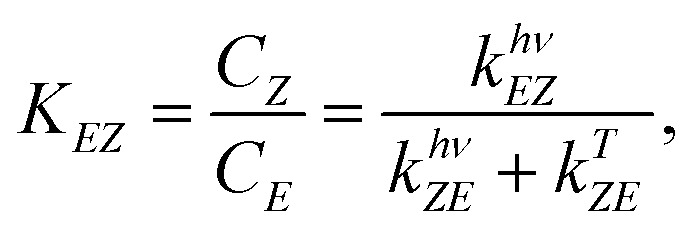
where *C*_*Z*_ and *C*_*E*_ are the concentration of the *Z*- and *E*-isomer; *k*^*λ*^_*EZ*_ the rate constant of the interconversion of *E* to *Z* photochemically through illumination with light of the wavelength *λ*; *k*^*λ*^_*ZE*_ the rate constant of the corresponding photochemical backreaction and *k*^*Δ*^_*ZE*_ of the thermal backreaction.^84^ Generalising, a rate constant of a given photoreaction of the substrate *i* to photoproduct *j* is dependent on various parameters:

5 *k*^*λ*^_*ij*_ = *f*(*E*_PF_(*λ*, *x*, *y*, *z*), *ε*_*i*_(*λ*), *φ*_*ij*_(*λ*), *d*, *μ*_a_(*λ*,*t*)),

where *E*_PF_ is the photon flux, which is proportional to the irradiance modelled; *ε*_*i*_(*λ*) molar absorption coefficients; *φ*_*ij*_(*λ*) is the quantum yield of the interconversion *i* → *j* through illumination with light of the wavelength *λ*, *d* the path length and *μ*_a_(*λ*, *t*) the total yet time-dependent absorption at a given wavelength.

An accurate interconnection of biophotonical Monte Carlo simulations with photokinetics holds a variety of challenges: foremost, there is a lack of experimental data quantifying the molecular properties of the photodrug. Besides, the photoreaction changes the total absorption coefficient within a cuboid over time. Multiwavelength models add a level of complexity to the modelling. Furthermore, it is unclear if the *ex vivo*-determined data concerning the molecular properties holds up in an *in vivo* environment.

However, combining the modelled photostationary state with a concentration profile and pharmacodynamic data, for our example, the half maximal inhibitory concentration IC_50_, will allow for mapping the inhibited and unaffected cuboids. In the first approximation and to establish the model, a constant concentration profile of photodrug could be assumed. Later, the concentration profile will require more refinement using pharmacokinetic data. This includes the data to quantify liberation and distribution, i.g., diffusion from the probe into the tissue, as well as metabolism and excretion, removing the photodrug from the side.

In summary, we made the first step into the computational modelling of *in vivo* photopharmacological inhibition by setting up a high-resolution rat model for the Monte Carlo simulation of light propagation into brain tissue. Moreover, we laid out a path towards a more comprehensive model.

## Conclusion

Photopharmacology can be a potent method to modulate neural activity. However, its practical application requires a broad combination of expertise. Here, we discussed the state of the art, highlighted current challenges and relevant technologies and presented new results.

The combination of simultaneous electrophysiological measurements of neuronal activities, drug delivery with light-driven de/-activation of an inhibiting photodrug represent an intriguing perspective for a future application in treating epilepsy. The here presented original results give a first projection of the validity of this concept. They motivate us to pursue exploring the demands for the application of photopharmacology.

## Author contributions

J. G. acquired funding and conceptualised as well as supervised the project. He design-engineered and fabricated the implants and contributed to the investigation and the corresponding data curation. He adapted the MCmatlab, visualised the data and wrote the original draft. T. E. N. contributed into investigation, data curation and visualisation. A. C. L. acquired funding. He developed the *in vivo* methodology and led their investigation. He visualised the data and revised the original draft. A. K. H. developed MCmatlab, helped to adapt it and revised the manuscript. G. G. M. acquired funding and conceptualised the project. He reviewed the manuscript.

## Data availability

The data supporting this article have been included as part of the ESI.† The MATLAB script is available on GitHub (https://github.com/ankrh/MCmatlab).

## Conflicts of interest

There are no conflicts to declare.

## Supplementary Material

TB-012-D4TB01117A-s001
