# Supplementary material for: In vivo photopharmacological inhibition of hippocampal activity via multimodal probes – perspective and opening steps on experimental and computational challenges
Source: J Mater Chem B. 2024 Aug 15;12(39):9894–904. doi: 10.1039/d4tb01117a (PMC11348833; doi:10.1039/d4tb01117a)
Supplement: TB-012-D4TB01117A-s001 [file TB-012-D4TB01117A-s001.pdf]

Supplementary Information (SI) for Journal of Materials Chemistry B.
This journal is © The Royal Society of Chemistry 2024

Electronic Supplementary Information for

In vivo Photopharmacological Inhibition of Hippocampal Activity via Multimodal Probes – Perspective and Opening Steps on Experimental and Computational Challenges

Johannes Gurke,* Alejandro Carnicer Lombarte, Tobias E. Naegele, Anders K. Hansen and George G. Maliaras

*Corresponding author: johannes.gurke@uni-potsdam.de

Contents

Methods and Materials 3

Chemicals, Materials and Components 3

General Methods 3

Implant design, fabrication and characterisation 4

Computation 5

MC modelling and optical properties of the modelled materials 5

In vivo experiments 6

Animal surgery 6

Electrophysiology recordings and drug delivery 7

Methods and Materials

Chemicals, Materials and Components

Solvents were purchased from Fisher Scientific and used without further purification. Parylene-C dimer was purchased from Specialty Coating Systems, Inc. Photo resin Young Optics Inc BV-007A and Proc3dure GR 1 for digital light processing 3D printing were purchased from IMakr and Formlabs Elastic FLELCL01 from Simply Rhino Limited and used without further modifications. 2in CZ-Si wafer, thickness = 279 ± 25 µm (100), negative photoresist AZ nLOF 2035 and positive photoresist AZ 10XT 520cP as well as developer AZ 726 MIF by Merck Performance Materials GmbH as well as TechniStrip® NI555 were purchased from MicroChemicals GmbH. Heraeus Clevios™ PH 1000 (PEDOT:PSS) were used. The DMSO (grad bioUltra), glycidyloxypropyl)trimethoxysilane (GOPS), dodecylbenzenesulfonic acid (DBSA) and ethylene glycol (EG) were purchased from Sigma Aldrich and used without further purification. Photodrug **1** was synthesised as reported[S1], dissolved in small amount DMSO and dissolved in saline. M0.6x5 mm pan head machine screws (SNZS-S0.6X2-VA) were purchased from MISUMI Europa GmbH. Flex cables (five ways, 1 mm pitch, 68610514422) by Würth Elektronik eiSos GmbH & Co. KG were purchased from RS Components Ltd. 1/32'' Portex Fine Bore Polyethene Tubing by Smiths Medical International Ltd. was purchased from ThermoFisher. High resolution film photomasks were purchased from JD Photodata. High purity oxygen (grade N6.0), sulfur hexafluoride (N3.0), tetrafluoromethane was purchased from BOC. Bi-colour SMD-LED chip with dome lens manufactord by Kingsbright (Part No. KPBD-3224QBDSEKC) were purchased from RS Components.

General Methods

Chemical vapor deposition of PAC was conducted with a Speciality Coating Systems PDS 2010 labcoter 2. Digital light processing (DLP) 3D prints were conducted with an Asiga MAX X UV385 using a for Formlabs Elastic FLELCL01 and Young Optics Inc BV-007A. Filament-based 3D prints were manufactured with an Ultimaker 3. Spin coating was conducted with a Spin coater POLOS 200. Photolithography was conducted with the Karl Suss MA6/BA6 Mask Aligner and e-beam physical vapor deposition (PVD) with an K J Lesker PVD 75 E-beam Evaporator. Reactive ion etching was conducted with a Plasma Pro 80 RIE. For positioning and placing procedures a Fineplacer® Pico ma by FineTech GmbH was used. Optic microscopy was conducted with a Nikon SMZ1270 equipped with a Nikon DFK NME 24UJ003, while for scanning electron microscopy (SEM) a Hitachi TM4000Plus tabletop microscope was used. For washing procedure incl. ultra-sonication an Bandelin sonorex digiplus DL255H has been used. Dry and tempering procedures were conducted in a Fistreem Vaccum oven

[S1] http://dx.doi.org/10.1039/C6SC01621A

equipped with a KNF N840 labport or a Fisherbrand 65K Oven 230V. Surface activations were conducted with a Diener electronics plasma surface technology Zepto RIE equipped with an Agilent IDP-7 Dry Scroll Pump or with a Diener electronics plasma surface technology Femto. Ultraviolet light curing was conducted with an Analytic Jena UVP Crosslinker CL-1000L. Deionized water was received from the water purification system Millipore RiOs -DI® 3UV. For drop-on-demand inkjet printing a Meyer Burger PiXDRO LP50 equipped with a JBinstruments printhead assembly for Fujifilm Dimatrix Cartridges (DMC-11610) were used. Layer thickness measurements were conducted with Dektak XT Profilometer by Brucker. Low level electrical characterisations of circuits were conducted with a Fluke 117 true rms multimeter. For electrochemical impedance spectroscopy a Metrohm Autolab PGSTAT128N was used in a three-electrode configuration, equipped with the Ag/AgCl electrode ZA006 AS RE-1B by ALS Co., Ltd as reference electrode and an Metrohm Platinum-wire electrode as counter electrode. A sinusoidal voltage input of amplitude 10 mV at different frequencies ranging from 1 Hz to 100 kHz was used. All computer aided design were conducted with Autodesk Inventor Pro2020. Light irradiance was determined using AvaSpec-ULS2048CL-EVO Fibre Optic Spectrometer (Part No. AvaSpec-ULS2048CL-EVO-RS-UA), equiebt with a 50mm Integrating Sphere, for light measurements (Part No. AvaSphere-50- IRRAD) by Avantes, purchased from Anglia Instruments. The spectrometer was calibrated using a *NIST Calibrated Halogen Lamp (*Part No. AvaLight-HAL-CAL-ISP50-Mini) by Avantes.

Implant design, fabrication and characterisation

Implant design and fabrication has been conducted as reported.[S2]

The 3D printed socket with integrated alignment (Fig. 1 ⑦and ⑧) were printed with Proc3dure GR 1 resin, using a DLP printer and printing parameters as provided by Asiga. The device was assembled manually. The LEDs were wired separately from the recoding electrodes and battery run.

Electrodes were examined for operativeness prior to surgery and impedance was measured upon in vivo experiments. Irradiance of the blue and red-light LED were characterised using an integrating sphere. Drug delivery was examined for operativeness prior to surgery.

[S2] https://doi.org/10.1039/D1MH01855H

Computation

The Monte Carlo simulation was conducted using the MatLab R2023a software package. As a source of the anatomy and geometric base, the segmented Waxholm Space Atlas of the Sprague Dawley Rat Brain[S3] has been combined with 3D data set of a skull [S4] and vascular blood system.[S5] The digital brain segments and data of the vascular system were converted into highly detailed .stl meshes with 3D slicer. The stl meshes were simplified using a quadric-based edge-collapse strategy, reducing the overall number of faces to 100.000.[S6] For that purpose MeshLab 64 bit v2023.12 program package by Visual Computing Lab has been used.[S7] If not state otherwise, the biological tissue was modelled using the generic tissue approach, where five parameters, i.e. blood content (B), blood oxygen saturation (S), water (W), melanin (M) and fat content, are used to quantify the absorption coefficient (μ_a) of tissues (equation 12 in Ref.[S8]). To quantify the reduced scattering coefficient of tissues the reduced scattering coefficient (μ'_s), fraction of scattering due to rayleigh scattering (f_{Ray}), scattering power for Mie scattering (b_{Mie}) and scattering anisotropy (g) have been used (equation 2 in Ref.[S8])

An LED light source for the MC simulations where modelled accosting to the product data sheet as a radial-factorizable beam with a top-hat radial focal plane intensity distribution and radial focal plane of 0.05 cm, a Cosine (Lambertian) Radial angular intensity distribution and Radial Width of the Angular Intensity Distribution of π/15 rad. The MC simulations resulted in a 3D Array of normalised fluence rate (Wcm^{-2} per W.incident). The input power of the bicolour LED was taken as reported in the product data sheet: 58.5 mW (460 nm) and 33.6 mW (610 nm).

[S3] https://doi.org/10.21203/rs.3.rs-2466303/v1
[S4] https://doi.org/10.1109/NER.2013.6696077
[S5] https://doi.org/10.1038/s41598-020-61656-1
[S6] https://doi.org/10.1145/258734.258849
[S7] https://doi.org/10.2312/LocalChapterEvents/ItalChap/ItalianChapConf2008/129-136
[S8] https://doi.org/10.1088/0031-9155/58/11/R37

MC modelling and optical properties of the modelled materials

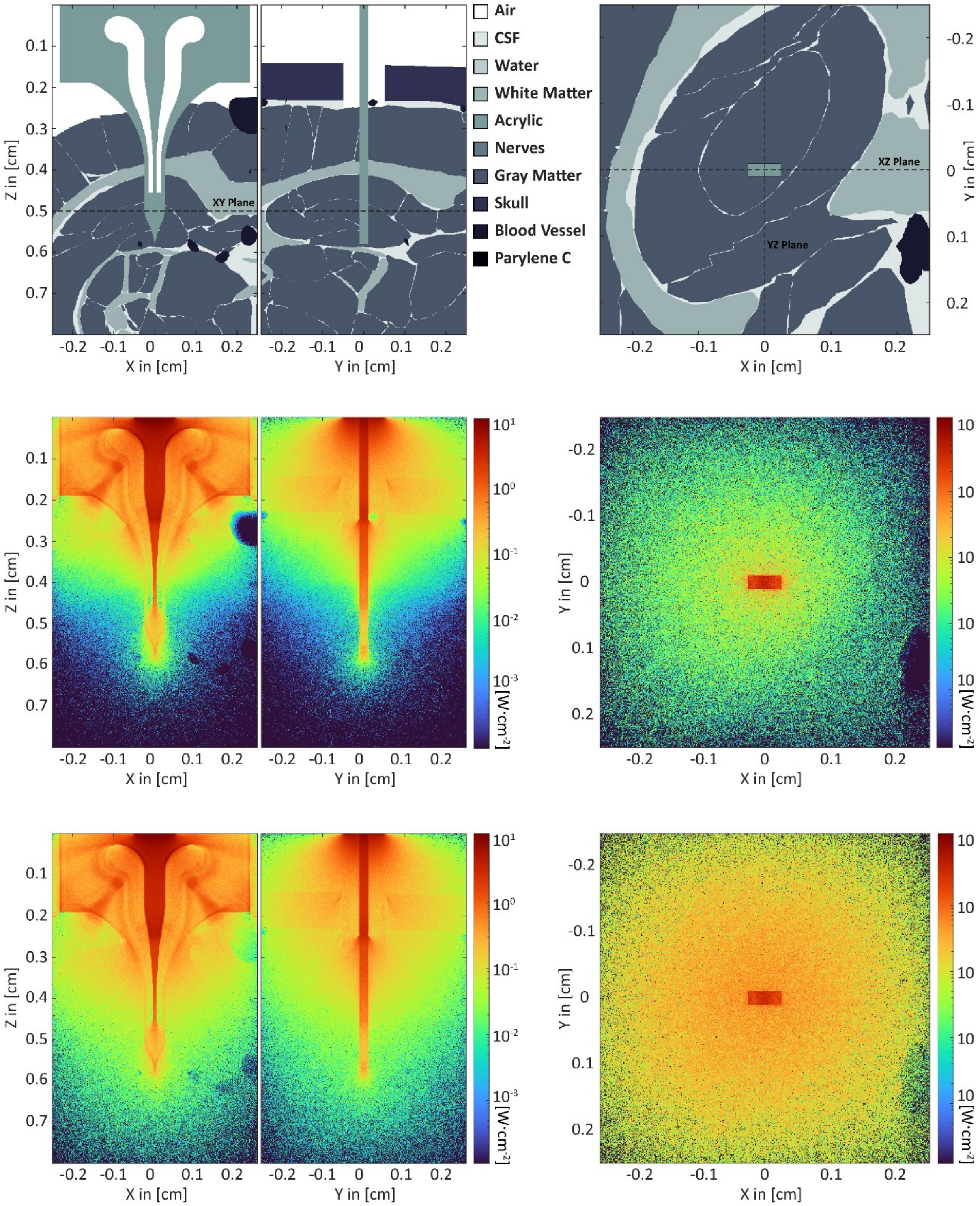

Figure S1 Geometric illustration of the digital rat brain model with the probe (top) and local irradiance maps for 460 nm (mid) and 610 nm (bottom) calculated via high resolution Monte Carlo simulations using 4x4x8 µm³ cuboids in W·cm^{-2}.

Media Properties Cerebrospinal fluid Ref[S9]

μ_a = 0.04 cm^{-1}

μ_s = 3 cm^{-1}

g = 1

n = 1.333 Ref[10]

Media Properties White Brain Matter, Ref[S8]

S = 59.0/100

B = 3.05/100

W = 0

F = 0

M = 0

aPrime = 31.0

fRay = 0.82

bMie = 0.000

g = 0.95

n = 1.4

Media Properties Gray Matter, Ref[S8]

S = 59.0/100

B = 3.05/100

W = 0

F = 0

M = 0

aPrime = 13.3

fRay = 0.36

bMie = 0.000

g = 0.95

n = 1.4

Media Properties Nerve, Ref[S8]

S = 59.0/100

B = 3.05/100

W = 0

F = 0

M = 0

aPrime = 25.9

fRay = 0.00

bMie = 1.156

g = 0.95

n = 1.4

Media Properties Skull

mua = 1.3 cm^{-1} Ref[S11]

aPrime = 9.7, Ref[S9]

fRay = 0.04, Ref[S8]

bMie = 0.116

g = 0.91

n = 1.564, Ref[S11]

Media Properties Parylene C

μ_a (462 nm) = 3.988 cm^{-1} Ref[S12]

μ_a (610 nm) = 1.275 cm^{-1} Ref[S12]

n = 1.65 Ref[S13]

Media Properties Clear, 3D printed acrylic resin.

μ_a (462 nm) = 1 10^{-8} cm-1

μ_a (610 nm) = 1 10^{-8} cm-1

N = 1.524 Ref[S14]

For μ_s, and g of Parylene and Acrylic Resin were no experimental data available. For Parylene they strongly dependent on the deposition method and conditions.

[S9] https://doi.org/10.1364/ao.45.004747

[S10] https://doi.org/10.1088/0031-9155/58/14/5007

[S11] https://doi.org/10.1155/2008/267867

[S12] https://doi.org/10.1364/OME.427952

[S13] https://doi.org/10.1116/1.1591744

[S14] https://doi.org/10.1364/OME.438040

In vivo experiments[S15]

Animal surgery

All experimental procedures were performed in accordance with the UK Animals (Scientific Procedures) Act 1986. The implantation protocol and in vivo experiments were conducted as established prior.[S2]

"~250 g Sprague Dawley rats (Charles River UK) were anaesthetised using isoflurane (2.5% in oxygen), and mounted on a stereotaxic frame. Their body temperature was monitored and maintained using a thermal blanket. A 1.5 mm^2 window was drilled into the skull, the dura carefully dissected, and the brain exposed. A hybrid fabricated probe was mounted onto the frame and gently lowered into the hippocampus CA1 (final coordinates for the tip of the probe: -4 mm anterio-posterior, 3 mm lateral, 3 mm depth).

For electrophysiology recordings (electrophysiology recordings and drug delivery), a stainless steel screw was drilled into the CSF above the cerebellum to act as a recording ground. Isoflurane anaesthetic was lowered to 1.25 % during and prior to recordings."[S2]

Electrophysiology recordings and drug delivery

"The hybrid fabricated probe was connected to electrophysiology recording hardware (RHS Stim/Recording headstage and controller, Intan Technologies), with the cerebellar screw acting as a ground. Voltage signals were recorded and amplified (X192), bandpass filtered between 1 Hz and 7.5 kHz, and digitised at a 30 kHz sampling rate. To stimulate interictal-like activity, the RHS Stim/Record ground was connected to one of the electrodes in the hybrid fabricated probe. This formed a stimulating pair with one other electrode in the probe, locally stimulating the hippocampal tissue. The two most distant electrodes (bottom left and top right - Figure 1) were chosen to form this pair. Stimulation was carried out over 300 ms at 500 uA and 250 Hz (1 ms per pulse phase, followed 2 ms of interpulse period)."[S2]

Local drug delivery was carried out by manually slowly injecting photodrug solution (20µL, 3.05 µM photodrug **1**, L-arginine 1 uM in saline with 1% DMSO) over 30 s through one of the microfluidic channels of the hybrid fabricated probe. Blank solution without photodrug was prepared and injected, too, showing no effect on the hippocampal activity. The brain was illuminated 60 seconds prior to electric stimulation.

“For analysis, data was notch-filtered to remove mains noise and band-pass filtered between 1 and 400 Hz. Spikes were identified and counted when they had an amplitude larger than three standard deviations above the baseline using a custom-written script. Electrophysiology data analysis was carried out using Spike2 software (Cambridge Electronic Design, UK, v9.04b).”[S2]

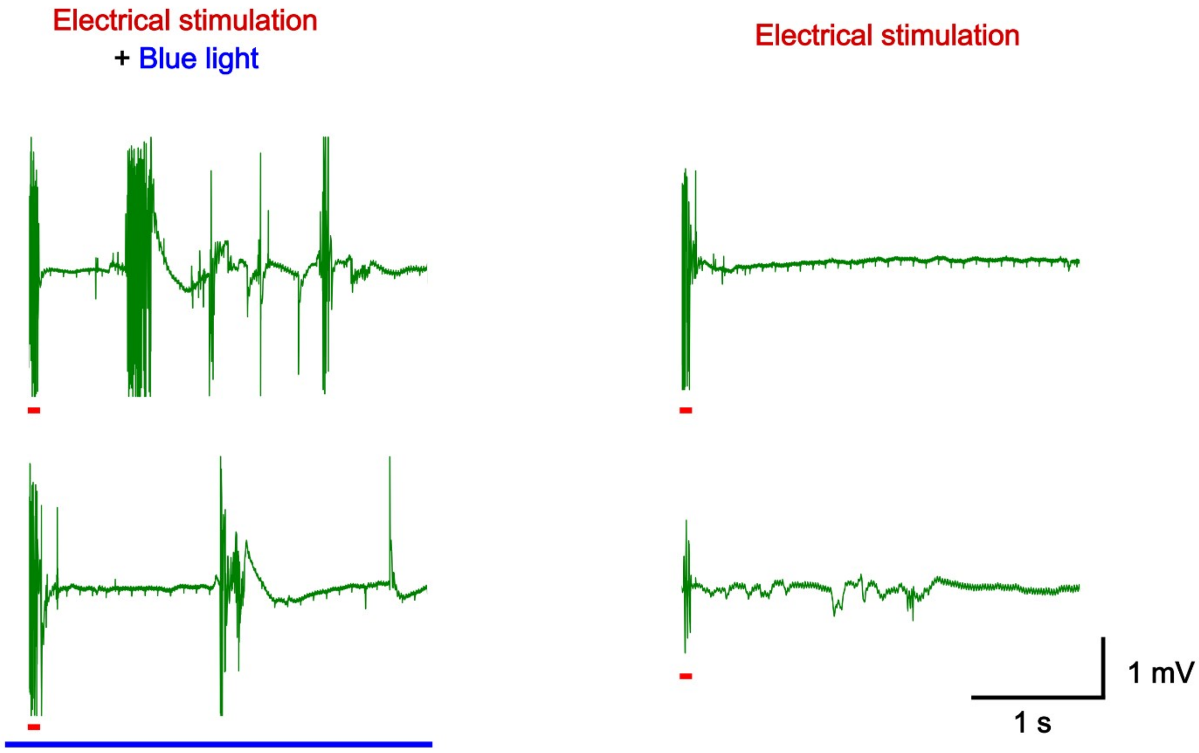

Figure S2. Additional recordings of hippocampal activity in response to electrical stimulation in the presence of photodrug 1 deactivated by blue light (left), or photodrug 1 in its active form (right), using multimodal probes. Seizure-like activity can be seen in the presence of the inactivated MPQX.

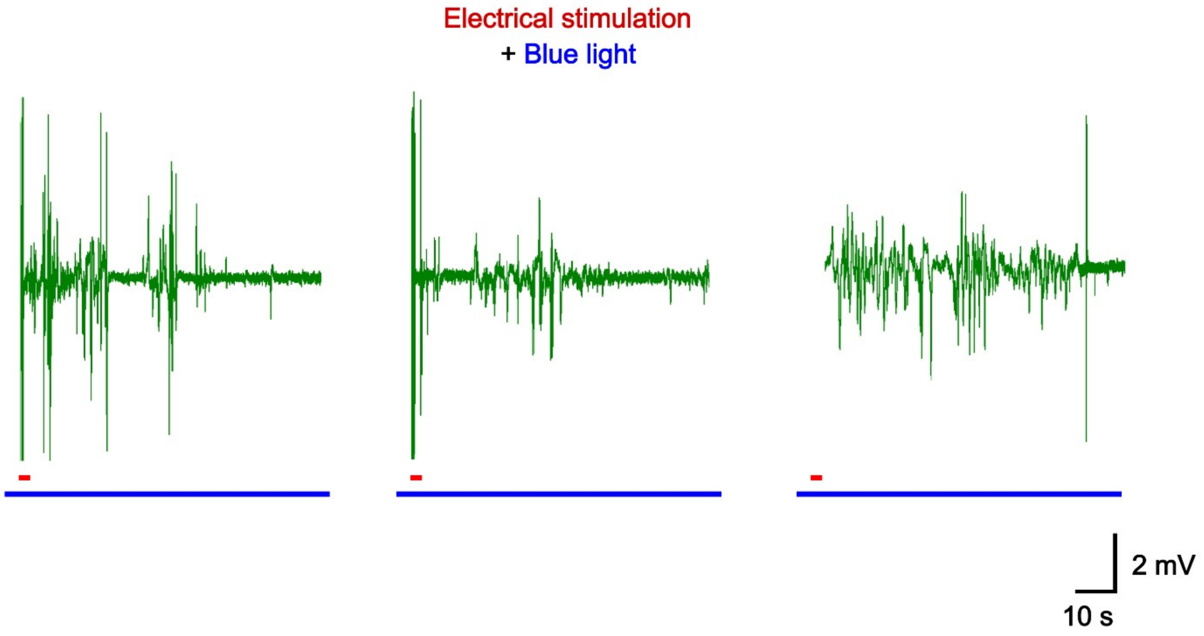

Figure S3. Blank experiment: Recordings of hippocampal activity following electrical stimulation while illuminated by blue light but in absence of photodrug 1, using multimodal probes. Seizure-like activity is elicited in response to electrical stimulation, indicating that the light delivered itself does not inhibit this response.
